# Novel low shear 3D bioreactor for high purity mesenchymal stem cell production

**DOI:** 10.1371/journal.pone.0252575

**Published:** 2021-06-16

**Authors:** Andrew B. Burns, Corinna Doris, Kevin Vehar, Vinit Saxena, Cameron Bardliving, Parviz A. Shamlou, M. Ian Phillips

**Affiliations:** 1 Keck Graduate Institute of Applied Life Sciences, Claremont, California, United States of America; 2 Sepragen Corporation, Hayward, California, United States of America; 3 Jefferson Institute for Bioprocessing, Jefferson University, Philadelphia, Pennsylvania, United States of America; Michigan Technological University, UNITED STATES

## Abstract

Bone marrow derived human Mesenchymal Stem Cells (hMSCs) are an attractive candidate for regenerative medicine. However, their harvest can be invasive, painful, and expensive, making it difficult to supply the enormous amount of pure hMSCs needed for future allogeneic therapies. Because of this, a robust method of scaled bioreactor culture must be designed to supply the need for high purity, high density hMSC yields. Here we test a scaled down model of a novel bioreactor consisting of an unsubmerged 3D printed Polylactic Acid (PLA) lattice matrix wetted by culture media. The growth matrix is uniform, replicable, and biocompatible, enabling homogenous cell culture in three dimensions. The goal of this study was to prove that hMSCs would culture well in this novel bioreactor design. The system tested resulted in comparable stem cell yields to other cell culture systems using bone marrow derived hMSCs, while maintaining viability (96.54% ±2.82), high purity (>98% expression of combined positive markers), and differentiation potential.

## Introduction

Stem cells are a major component of regenerative medicine because of their potential to cure chronic diseases and regenerate organs [1]. They are defined by their abilities to self-renew and differentiate into functional cell types. One promising stem cell is the mesenchymal stem cell, which can differentiate into many useful cell types for regenerative medicine, including osteocytes, adipocytes, chondrocytes, myocytes, and cardiomyocytes [2]. Human Mesenchymal Stem Cell (hMSC) derived cell therapies are currently under clinical trials for cardiovascular, neurologic, bone and cartilage, lung, kidney, liver, and autoimmune diseases [3]. They also show immunotolerant and immunomodulatory properties in allogenic transplants [2,4]. However, hMSC harvest can be invasive, results in relatively low yields, and is painful, making therapies difficult and expensive [5]. A possible solution is to culture the stem cells and produce large quantities through bioprocessing, yielding many therapeutic dosages from one harvest.

The challenges of creating clinically relevant dosages lie in both the number of cells needed for a successful therapy, and the sensitive nature of hMSCs. For example, it is estimated that a therapeutic dose between 10^6^ and 15x10^6^ stem cells per kilogram is needed to treat myocardial infarction due to apoptosis, off-target differentiation, and cell attrition after implantation [6–12]. To supply such large quantities, researchers have investigated scalable bioproduction of hMSCs. However, conventional scalable bioreactor culture procedures are difficult to implement for hMSC culture, as they are anchorage-dependent and sensitive to both mechanical and chemical stresses [13–18]. Bioreactor culture conditions must be tailored to this delicate cell type to maintain stemness during bioproduction. Reactor designs vary, but fall into three general categories: stacked two-dimensional surfaces, microcarrier or aggregate based, and fixed bed reactors. Table 1 shows a summary of cell yield and reported values of various cell culture systems. A drawback to some of these systems is that their method of fluid handling can lead to high shear stress. Furthermore, feasibility studies are very expensive and scale down models do not exist for all the listed systems. Because of this, we developed a scale-down model of a novel culture system designed for anchorage dependent stem cell culture.

**Table 1:** Comparison of culture systems.

Name	Type	Classification	Vendor	V (mL)	SA (cm^2^)	SA:V	Cell type	SC/mL x10^6^	SC/cm^2^ x10^4^	Total SCs x10^6^	Td (hr^-1^)	Shear (dyne cm^-2^)	Source
Packed Bed	PDMS Matrix	Immobilized	-	110	2,800	25.5	hP-MSC	0.509	2.00	56	30.2	1–5	[19]
Quantum	Hollow Fiber	Immobilized	TERUMOBCT	1,440	21,000	14.6	hAd-MSC	0.167	1.14	240	34.1	0.3–0.7	[20–23]
Mobius	STBR	Suspension	Millipore Sigma	50,000	300,000	6	hBM-MSC	2.00	1.67	5,000	54.0	2–40	[21,24,25]
Appliflex	Wave Bag	Suspension	Applikon	1,500	7,360	4.91	hAd-MSC	0.190	3.87	285	31.2	0.1–0.5	[6,26]
Mag 3	Paddle	Suspension	PBS	3,000	-	-	hBM-MSC	1.90	-	5,700	63.0	-	[27]
Xpansion Multiplate	Parallel Plate	Immobilized	Pall	1600	6,120	3.83	hAd-MSC	0.111	5.4	334	34.1	0.1	[28,29]
iCellis	Random Fiber Matrix	Immobilized	Pall	1000–5000	40,000	40	hBM-MSC	2.93	16	-	67.2	1–5	[25,30,31]
Novel System	Lattice	Immobilized	-	20	122	6.1	hBM-MSC	0.2	2	1.8	82	0.0029	-

Abbreviations: Volume (V), SA Surface Area (SA), Stem Cells (SC), Doubling Time (T_d_), Mesenchymal Stem Cell (MSC), Human Bone Marrow (hBM), Human Adipose Derived (hAd), Human Placental (hP).

Volume, available surfaces areas, cell types used, total stem cell yield normalized to volume and surface area, total overall yield, doubling time, and reported shear stress of various bioreactors for MSC culture.

Here we investigate a scaled model of the Express bioreactor by Sepragen. The system uses gravity and capillary action to drive media through a cellulosic-based honeycomb matrix suspended out of the media. Cells are captured in the matrix, where they proliferate. This impeller-free method of liquid handling creates a very thin, slow-moving layer of media over the matrix, allowing for excellent gas exchange and low shear. To reduce cost and have more control of testing, a small-scale system was made using the same liquid handling regime. The goal of this study was to investigate how this system would work specifically with shear sensitive hMSCs. To accomplish this, we designed a new culture area using a rigid polymer matrix to be compatible with anchorage-dependent cells specifically for the system. Conventional methods of creating 3D scaffolds, such as casting and molding, can provide a large culture area, but are labor intensive [32–35]. Because of this, and previous works, we propose 3D printing as a very controlled, rapid, and low cost alternative for rigid scaffold manufacturing [36,37].

The prototype system uses a rigid, porous 3D printed Polylactic Acid (PLA) lattice. PLA was chosen, as it is both biocompatible and biodegradable, and is a thermoplastic commonly used in 3D printing [38,39]. The facets of the lattice are designed to allow unimpeded media flow, while significantly increasing the surface area available for cell growth compared to conventional 2D culture methods. Computational Fluid Dynamics (CFD) was employed in designing the lattice, allowing rapid testing of shear forces experienced by cells within the scaffold. The final design consists of a 3D printed 400μm cross-fiber lattice, resulting in good media diffusion and very low shear stress. Small removable pieces are integrated into the lattice matrix, which are easily accessible for cell sampling and imaging. By combining this dynamic culture method with hypoxic conditioning, stem cell proliferation was significantly increased while maintaining the International Society for Cell and Gene Therapy (ISCT) definition of hMSC purity [40]. Furthermore, we found that cells cultured in this 3D, low shear system showed decreased doubling times compared to conventional flask-based culture. With this, we have successfully proofed a novel platform for small-scale bioreactor culture of shear sensitive adherent cells.

## Methods

### Stem cell culture

hMSCs were cultured according to guidelines provided by the American Type Culture Collection (ATCC). Briefly, cells were cultured in hMSC media (ATCC PCS-500-030) supplemented with the bone marrow derived hMSC bullet kit (ATCC PCS-500-041) at 37°C and 5% CO_2_ on T-75 treated tissue culture flasks. A ¾ media exchange was performed on day three, and cells were passaged at 80% confluency, usually on days six or seven. Cells were lifted using 0.25% trypsin and 0.53 mM EDTA solution (ATCC 30–2101) for regular passaging of T-75 flasks, and re-plated at 5,000 cells cm^-2^. Cell pelleting was performed by centrifugation at 270g for five minutes. A working cell bank was created from passage four hMSCs and stored in liquid nitrogen until use. Experiments using hMSCs were conducted on cells between passage five and nine. Doubling time and specific growth rate were calculated to compare culture success.


$$T_{d}=(T_{2}-T_{1})*\frac{\ln\left(2\right)}{\ln\left(\frac{q_{2}}{q_{1}}\right)}$$
**Eq 1:** Doubling time. Where T_d_ is the doubling time, T_2_ is the total time elapse of the run, T_1_ is the time of seeding, q_2_ is the final cell yield, and q_1_ is the initial cell seeding quantity.

$$\mu =\frac{ln(\frac{q_{2}}{q_{1}})}{\left(T_{2}-T_{1}\right)}$$
**Eq 2:** Specific growth rate. Where μ is the specific growth rate, T_2_ is the total time elapse of the run, T_1_ is the time of seeding, q_2_ is the final cell yield, and q_1_ is the initial cell seeding quantity.

### Oxygen tension studies

To induce low oxygen states in flask culture, cells were placed in a hypoxia chamber (Billups-Rothenberg) and gas flushed for six minutes at 5PSI with a flow rate of 10L min^-1^. Gas composition was mixed based on PSI. For reactor cultures, the mixed gasses were introduced at 100mL min^-1^ for five minutes to exchange the bioreactor head space and oxygen from the media. At first a tri-gas mixture of nitrogen, oxygen and carbon dioxide was used, but carbon dioxide was excluded from later bioreactor runs with no impact on yield and purity. Hypoxic gas was overlaid and capped for three days, at which point normoxic gas was reintroduced to the culture. The treatment period for 0% O_2_ was reduced to only one day due to cell loss.

### Bioreactor construction

**Fig 1:**
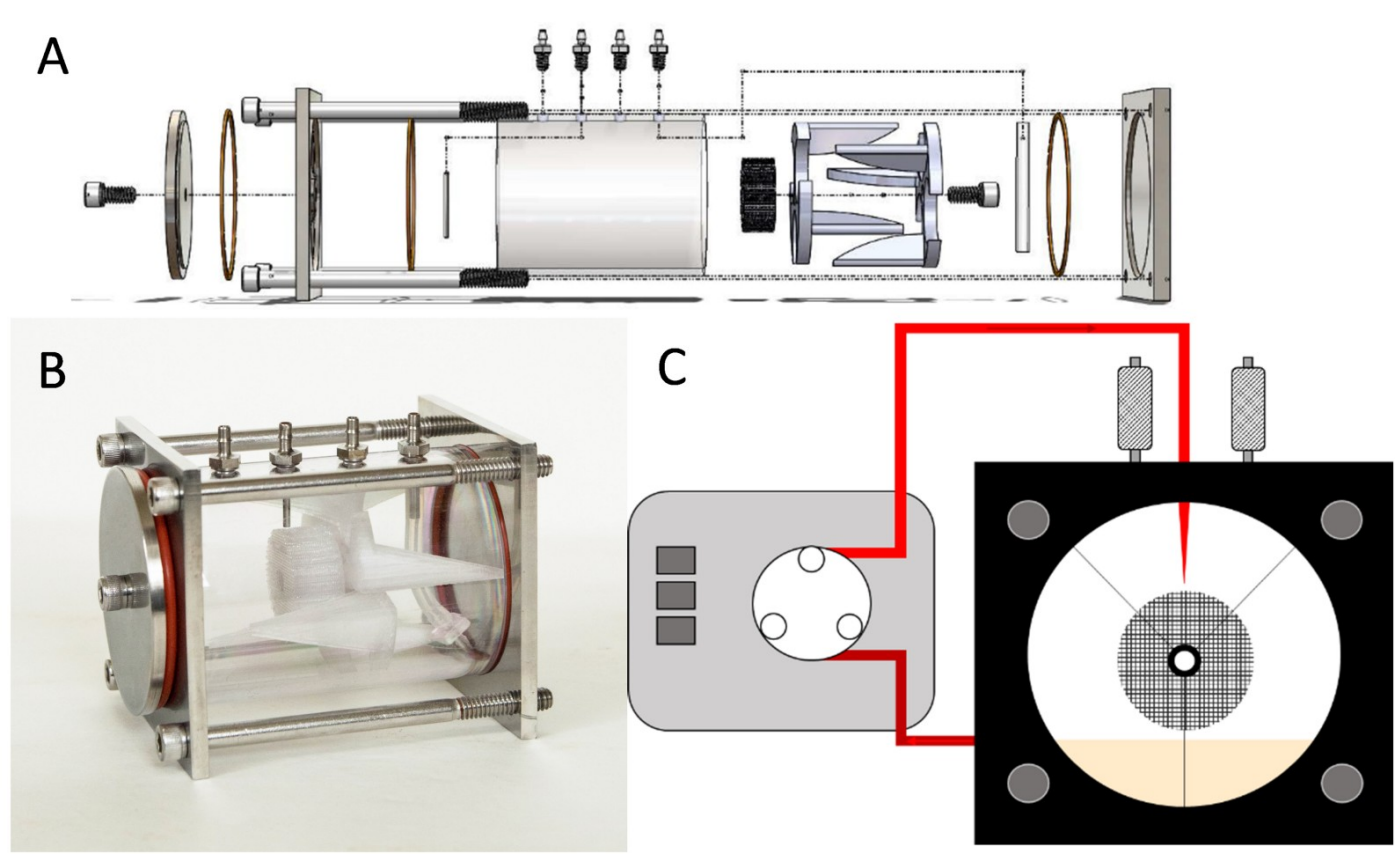
Schematic and picture of the bioreactor. A) Exploded diagram of components and how they are assembled. Orange O-rings seal the polycarbonate chamber against the stainless steel ends. The small sub-assembly in white is comprised of two identical 3D printed stabilizing pieces, held together against the 3D printed cylindrical culture matrix with a stainless steel thumb screw. B) Actual image of the assembled reactor. C) Cartoon front-on schematic of how lattice is suspended out of media and fluid is recirculated through the system.

The chamber of the reactor is made of a 90mm long polycarbonate tube with an ID of 57.15mm (2.25in) and an OD of 63.5mm (2.50in). Four 316 stainless steel barbed hose adapters are tapped into the top of the polycarbonate: two for media circulation and two for gas exchange through 0.2μm filters. Size 14 silicone tubing was used for the main liquid handing loop, with a 14-gauge Tygon Pharmed section for peristaltic pumping. The head plate and back plate are made of 316 stainless steel. An exploded diagram of the reactor components can be seen in Fig 1A. The interior reactor components include the 3D printed lattice matrix, which is suspended out of the media using two 3D printed brackets. The fully assembled bioreactor can be seen in Fig 1B. Overall it measures 100mm x 80mm x 80mm, with the cylindrical culture volume measuring approximately 218mL. Media pumped to the top of the lattice is perfused through the lattice design via gravity, as depicted in Fig 1C. This thin layer of flowing media provides gas exchange and nutrients to the cells. Gas control is highly tunable, as there is less liquid for gas to diffuse through to be available to the cells. The front plate has a pass-through port for access to the removable sampling scaffolds, allowing a means to monitor cell confluency. The minimum and maximum working volumes tested were 20mL and 30mL.

### Lattice design and bioreactor culture

The PLA lattice matrix was 3D printed using a PrintrBot Simple printer and Cura 3D (V3.2.1) printing software. A 0.4mm nozzle diameter was used to print the PLA scaffold. The lattice was constructed such that the smallest features are printable with a conventional 3D printer while allowing enough uninterrupted surface area for cells to culture into monolayers. For this extruder, the lower limit of resolution was 400 microns in the XY plane. The lumen between fibers was made to be the same width as the fiber itself. Also included in the design are two inserts for non-destructive means of visualization of cell confluence and viability via calcein staining. The system was fully assembled as a closed system and autoclave sterilized at 121°C for 15 minutes under a dry cycle. After sterilization, the matrix is washed and wetted with filtered and autoclave-sterilized 1x PBS (VWR VE404) and gelatinized with filtered and autoclave-sterilized 0.1% (W/V) gelatin (Fisher 9000-70-8) in MQ water, for 45 minutes at 37°C, or overnight at 4°C. The matrix was then rinsed with PBS to remove unbound gelatin, and cells were seeded at 2,500 cells cm^-2^. Approximate surface area was calculated using Solidworks (Waltham, MA) analysis function. To force cell attachment only within the lattice, the desired number of cells were resuspended first in a total of 2mL, as this volume was found to be the holding volume of the matrix. Cells were allowed to settle in the matrix for 45 minutes before starting the recirculation loop. Recirculation was run between 0.25mL min^-1^ and 0.50mL min^-1^. A range is noted because as the peristaltic tube relaxed during use, the peristaltic pump tended to speed up, slightly increasing the flow rate over time. 0.25mL min^-1^ was used as it was the slowest rate that still allowed complete matrix wetting. A ¾ media exchange was performed on day three, and cells were harvested on day seven using a lifting cocktail comprised of a 2:1 mixture of Cell Dissociation Buffer (CDB) (Gibco 13151014) and TrypLE-Express (Gibco 12604021). Cells were lifted by first aspirating the culture media and cycling 10mL of PBS through the system at 1.0mL min^-1^ to remove residual media. PBS was then aspirated, and a cell-lifting cocktail was added. The reactor was then cycled at 1.0mL min^-1^ for 15 minutes. Viability and cell counting were performed using trypan blue staining and a hemocytometer.

### Microcarrier culture in spinner flask

Cytodex-1 microcarriers were weighed and autoclaved at 121°C for 15 minutes. Microcarriers were then hydrated in hMSC media. Cells were seeded at 5,000 cells cm^-2^ in 50mL of media in a 250mL spinner flask (Wheaton). The spinner flask was set to 15RPM for 24 hours to allow hMSCs time to adhere to the microcarriers, after which agitation was increased to 30RPM and volume increased to 80mL. A ½ media change was performed on day three. Samples were drawn each day and fixed in 4.0% paraformaldehyde (PFA) for 15 minutes for imaging. Samples were prepared for cell counting via DRAQ5 (Abcam ab108410) staining in a 5.0mMol solution overnight. The culture was run for a total of seven days. On day seven, the microcarriers and media were collected and allowed to settle for 20 minutes. The supernatant was then aspirated, and microcarriers were washed twice with PBS. When the microcarriers resettled, TrypLE was added and the mixture was incubated for one hour to lift cells for counting and characterization.

### Confocal microscopy

Cells were cultured on lattice matrices in the bioreactor for seven days. Cells were washed using Ca^++^ and Mg^++^ PBS and fixed in place with 4.0% PFA for 15 minutes. Permeabilization was performed using 1.0% (W/V) Triton-X 100 in PBS for 30 minutes at 37°C. Cells were then washed and blocked in 1.0% (W/V) Bovine Serum Albumin (BSA) and 0.1% (W/V) Triton-X 100 in PBS for one hour at room temperature. Cells were then stained for 30 minutes with one drop mL^-1^ Phalloidin green (Invitrogen) and 1.0μL mL^-1^ DRAQ5 (final DRAQ5 concentration of 5.0mMol). Cells were then washed with Ca^++^ and Mg^++^ PBS and imaged on a Leica SP5 confocal microscope.

### SEM imaging

PLA matrices were washed and prepared for electron microscopy. Samples were attached to 0.5in slotted stages (TED PELLA 16111) using conductive double-sided copper tape. Samples were imaged at 2kV using a Hitachi SU-70 scanning electron microscope.

### Flow cytometry

hMSCs were cultured in experimental conditions and lifted with a 2:1 mixture of CDB and TrypLE-Express lifting cocktail to preserve cell surface markers. Cells were washed in PBS and placed in the lifting cocktail for 15 minutes. After neutralization with fresh media, cells were fixed in 4.0% PFA for 15 minutes, washed twice with PBS, and blocked for one hour at room temperature. Blocking solution consisted of 1.0% (W/V) BSA and 0.1% (W/V) Triton-X 100 in PBS. Cells were stained for the positive markers CD105 (Invitrogen MHCD10520) and CD73 (Abcam ab157335), and negative markers CD14 (Abcam ab91146) and CD19 (Abcam ab25510) at 1.0μL per 500,000 cells in 500μL following Abcam recommendations. Samples were run at 35μL min^-1^ on a BD Accuri C6 flow cytometer and analyzed using FlowJo (Ashland, OR). Unstained controls were used to gate cells. Fluorophore compensation was done using FlowJo and Fluorescence Minus One (FMO).

### hMSC Differentiation and staining

ATCC differentiation toolkits for osteocyte (PCS-500-052) and adipocyte (PCS-500-050) differentiation were used. hMSCs were seeded at 12,000 cells cm^-2^ and cultured for three days in hMSC media following ATCC Toolkit protocols for both adipocyte and osteocyte differentiation. On day 20, cells were washed with Ca^++^ and Mg^++^ free PBS and fixed by 4.0% PFA at room temperature for 15 minutes. Samples were stained following respective protocols explained below and visualized on a phase-contrast Olympus IX microscope.

Chondrocyte induction was performed according to a combination of ATCC protocols and previous research. Briefly, hMSCs were lifted from the reactor using the mentioned lifting cocktail and counted. Cells were resuspended in chondrocyte differentiation toolkit (ATCC PCS-500-051) at 1.25x10^6^ cells mL^-1^. 200μL of cell-laden media was added to 15mL polypropylene Falcon tubes and centrifuged at 270g for five minutes to pellet the cells. The tubes were then placed into the incubator with loosened caps for 24 hours without resuspending the cell pellet. After 24 hours the cell aggregate was gently resuspended via pipetting. Media was then changed every third day for 21 days, at which point the aggregates were sliced into 8.0μm thick samples using a Microm HM 500 cryostat and OTC compound (Tissue Tek 4583), and place onto glass slides. The sample slides were then washed gently in DI water and overlaid with Alcian blue stain for 30 minutes. The samples were rinsed with DI water, then washed with 3.0% (V/V) glacial acetic acid solution in MQ water to remove excess dye. Samples were then gently rinsed again with DI water and visualized.

Alizarin Red stain was used to stain osteogenic differentiation of hMSCs. After fixation cells were washed twice with MQ water, then stained for 15 minutes at room temperature. Samples were then washed three times with MQ Water and visualized.

Oil Red O was used to stain adipogenic differentiation of hMSCs. A working solution was prepared by mixing 3.0mL of Oil Red solution (#O-1391, Sigma) with 2.0mL of MQ water immediately before staining. Cells were covered with oil red working solution and stained for 30 minutes at room temperature. Samples were then washed twice with MQ water and visualized.

### Computational fluid dynamic modeling

**Fig 2:**
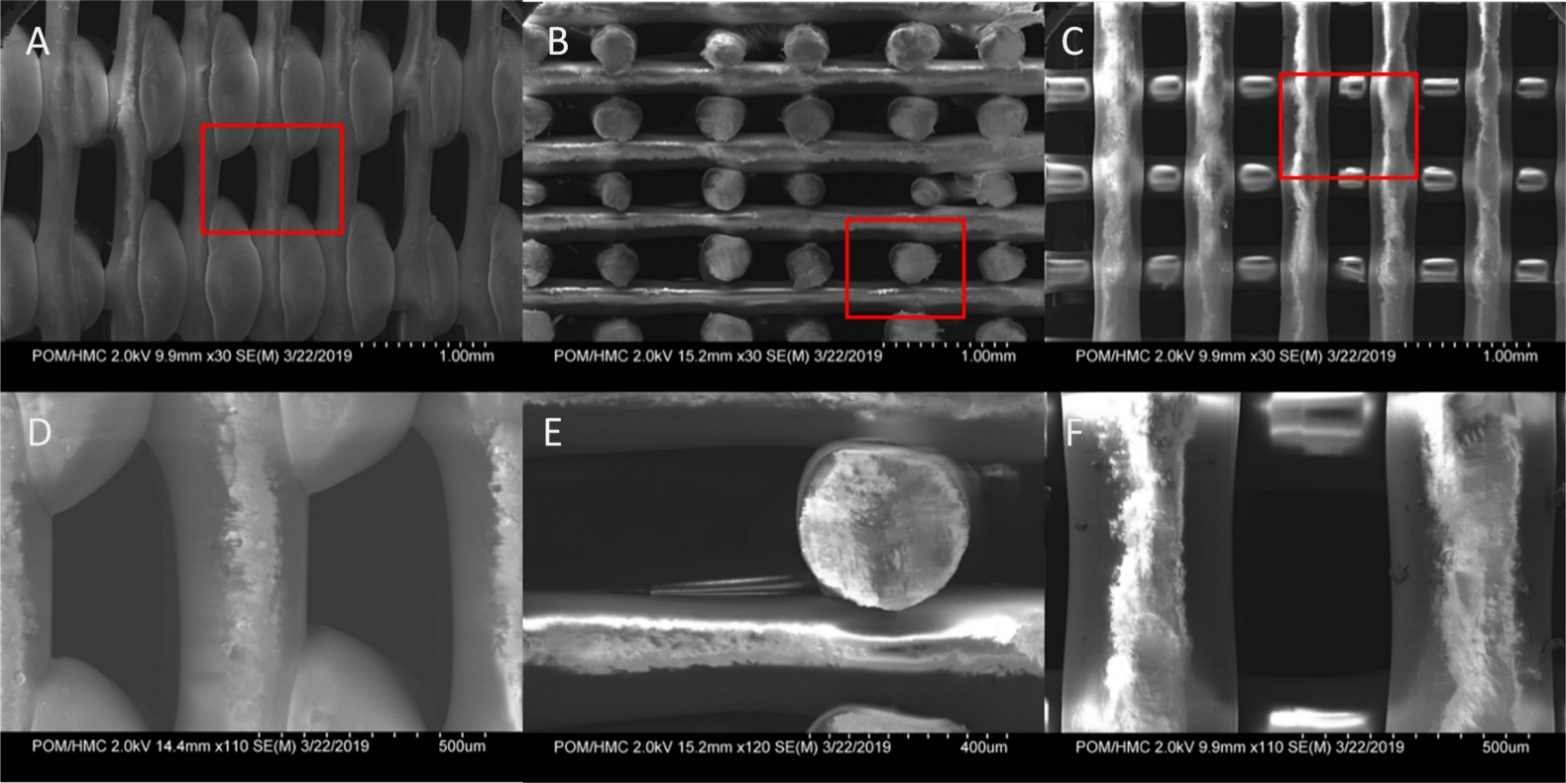
Scanning Electron Microscopy (SEM) images of 3D printed PLA. First row shows the following orientations of the lattice: A) exterior side, B) cut interior, and C) top-down view of PLA lattice. The second row shows higher zoom of images in top row: E) exterior side, F) cut interior, and G) top-down view. Red squares denote the zoomed field of view.


The system was simulated using ANSYS FLUENT 2021 R1 (ANSYS Inc., Canonsburg, PA). The goal of this model was to calculate shear stress in the system, and rapidly test shear across multiple flow rates. First, SEM images of the scaffold (Fig 2) were used to create a working geometric representation of the matrix to model the system in FLUENT. Fibers appeared very uniform in geometry, with an oblong shape. Because of the sharp angles at the intersection of fibers, we modeled the fibers as squares in our two-dimensional model. Though the lattice is three-dimensional, the overall construction is the sum of many repeating units. Given this symmetry, we were able to break the simulation down into its most simple two-dimensional unit for modeling. This decreased computational time and increased the convergence of the model tested. The modeled fluid domain within the lattice is constructed of crossing 0.4mm square flow channels and includes inlet and outlet boundary conditions. The fluid domain was divided using a quadrilateral mesh with a mesh density of 56 elements mm^-2^. The simulation was solved using the pressure-based solver with an absolute velocity formulation and the multiphase Volume of Fluid model with water and air phases. Inlet velocity was calculated by taking volumetric flow over the diameter of the simulated inlet. We modeled the system assuming laminar flow because of the low velocities of fluid movement through the matrix. The SIMPLE pressure-velocity coupling scheme was used to run a transient model with a tracer, using a time step of 0.01s and 15 iterations per timestep for a total of 18,000 timesteps to model three minutes of run time. Momentum convergence was set to 10^−6^. This transient model was then compared to a dye tracer benchtop experiment to test the predictive accuracy of the model. The reactor was cycled at the desired operating flow of 0.25mL min^-1^, and a dye tracer was injected above the scaffold. Video was taken of the dye moving through the scaffold and compared to the CFD model. Images taken at one, two, and three minutes were compared to equivalent time points in the simulation. The model was in close agreement with the benchtop dye test in both velocity (7.5% difference in velocity), and dye infiltration throughout the matrix (S1 Fig). The model was then run in steady-state to create a profile of flow rate vs shear stress within the system. Shear stress was calculated using Eq 3 and the reported strain rate from the model.

τ=ηγ
**Eq 3:** Where τ is the shear stress, η is the viscosity of the liquid, and γ is the strain rate (s^-1^).

### Statistics

Graphs and statistics were done using Minitab 17 (Minitab Inc., PA). Error bars on graphs show two standard errors. Student’s two‐tailed t‐test was used to determine significance for two data sets. Significance of multiple data sets was performed via one-way ANOVA and Tukey test.

## Results

### Scaled bioreactor system

The aim of this work was to show high purity, high yield stem cell culture on a 3D biocompatible lattice. To accomplish this, a scaled model of the system of the Express bioreactor was engineered, conserving the main geometry of the reactor chamber and method of media handling. As stated in the introduction, PLA was used in lieu of cellulosic. This is because cells tended to grow in clusters on cellulosic fibers (S1 Fig). To accommodate the PLA lattice in an unobtrusive way, it was suspended using two 3D printed stands. A 316 stainless steel tip was added to the inlet to allow seamless flow from the recirculation loop to the lattice. From 3D modeling in Solidworks it was calculated that the 30mm diameter lattice used has a theoretical surface area of 225cm^2^. Each 30mm diameter repeating layer provides 23.5cm^2^, which equates to a 32-fold increase in surface area compared to a conventional flask.

### CFD modeling

**Fig 3:**
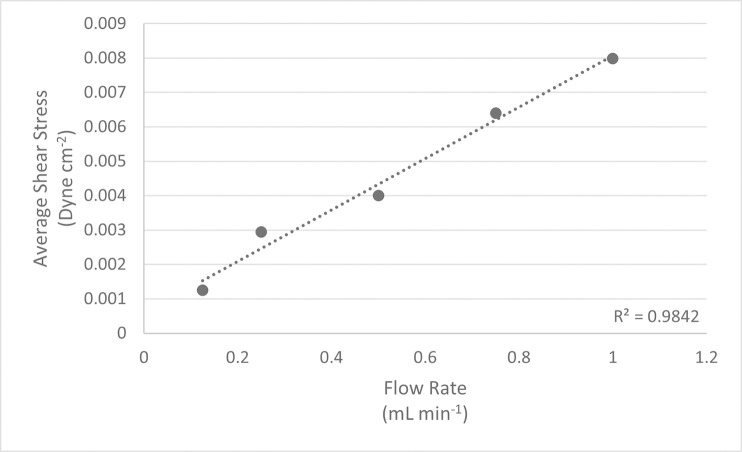
Inlet Flow Rate vs Average Wall Shear Stress. The lattice matrix was modeled in ANSYS and tested at various flow rates using Fluent. Strain rate was converted into shear stress using Eq 3.

**Fig 4:**
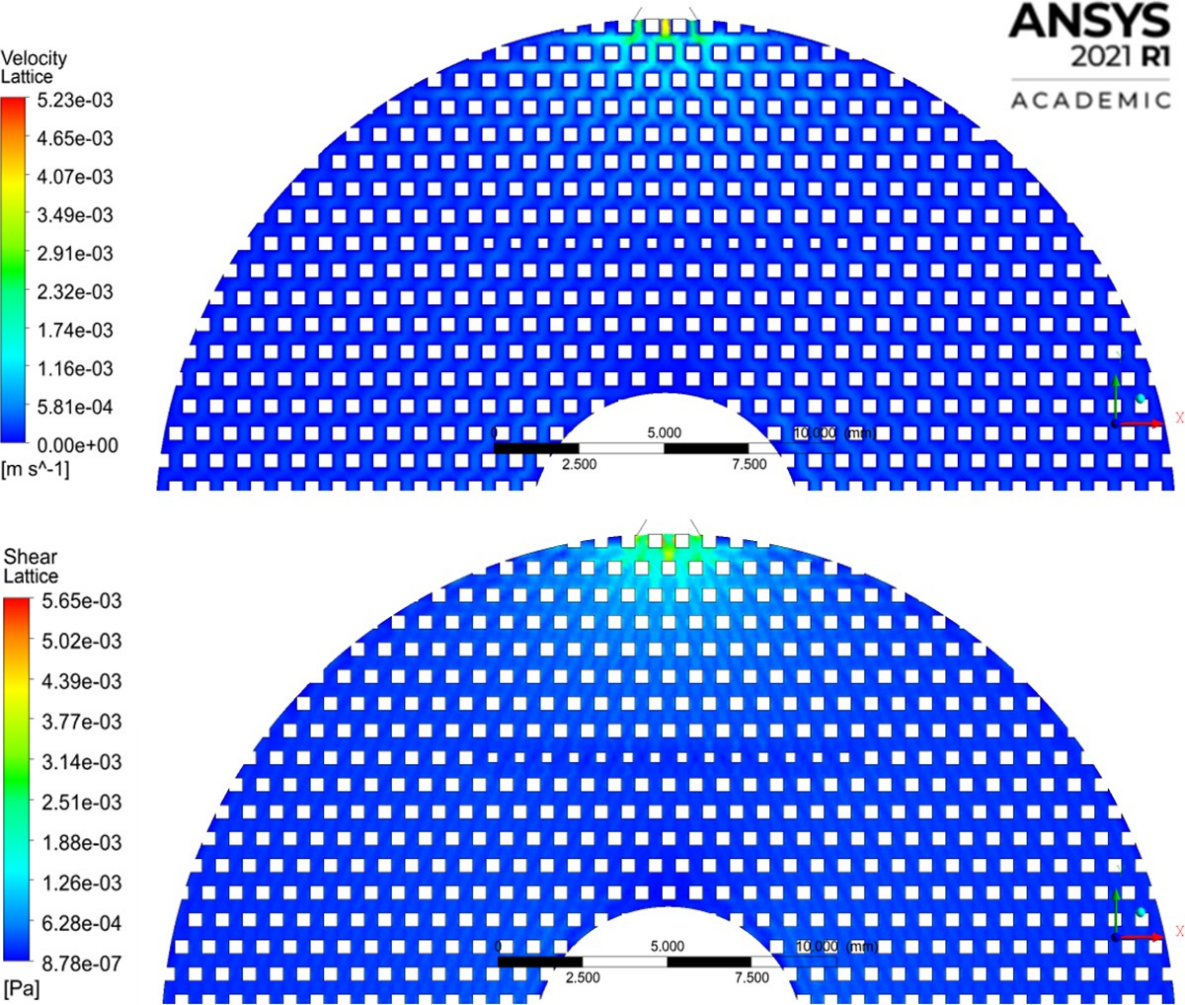
CFD modeling of lattice matrix. A) Velocity contour and B) shear stress at the inlet to the matrix. Maximum velocity of 0.00523m s^-1^ and maximum shear stress of 0.00565 Pa measured inside the lattice, excluding the interior of the inlet and outlet.

To quantify the range of shear stress within the system, we tested a range of flow rates to estimate shear stress vs flow rate using CFD (Fig 3). Shear stress increased linearly with fluid velocity. A flow rate of 0.25mL min^-1^ was used for stem cell culture because it kept the matrix well wetted while resulting in the lowest shear strain tested. At this flow rate, CFD modeling reported an average shear stress of 0.0029 dyne cm^-2^, and a maximum of 0.056 dyne cm^-2^. The area of highest shear stress was at the top center of the matrix insert, where the media was entering the lattice (Fig 4). Shear stress decreased throughout the body of the matrix as the fluid diffused through the pores. It then increased slightly as fluid was funneled toward the outlet at the bottom of the model, mirroring what occurred at the inlet.

### Spinner flask control

hMSC growth was also investigated on Cytodex-1 microcarriers in small-scale spinner flasks as a benchmark for suspension culture. Microcarriers have been well used in conventional suspension systems as a means of culturing adherent dependent cells in 3D. Performing this study allowed us to directly compare this technique to our lattice matrix. Static Polystyrene (PS) flask cultures (n = 6) showed an average doubling time and specific growth rate of 119.07hrs ±11.23 and 0.0062 hr^-1^ ±0.0013 respectively (S3 Fig). Cells cultured in spinner flasks showed an average doubling time of 113.60hrs ±23.75 and a specific growth rate of 0.0062hr^-1^ ±0.0013 (n = 3). Both are significantly longer (p = 0.002) compared to lattice reactor (n = 5) results, which had a doubling time of 81.96 ±4.96hrs and a specific growth rate of 0.00085hr^-1^ ±0.0005. Reactor results are discussed more thoroughly in the bioreactor section later.

### Cell viability on PLA lattice

Cell viability was compared between culture substrates and static vs dynamic cultures as previous studies with fibrous matrices exhibited increased cytotoxicity. Dynamic culture refers to cultures performed in the bioreactor, and static culture refers to control cultures performed in flasks. On day seven cells were enzymatically lifted, and viability was tested via trypan blue staining. PLA lattices were removed from culture wells to isolate only cells adherent to the PLA lattice. Dynamic PLA cultures from the bioreactor had an average viability of 96.54% ±2.82. Cells grown in dynamic bioreactor culture on PLA showed no statistically significant difference from static PLA culture plates, with an average viability of 96.76% ±3.84 (p = 0.98). Dynamic PLA showed no difference from static PS, which had an average viability of 95.13% ±1.07 (p = 0.45). Static PLA and PS showed no statistical difference in viability (p = 0.38). Therefore, PLA showed no detrimental effects on cell viability in either static or dynamic culture compared to control polystyrene flasks.

### Bioreactor dynamic culture

**Fig 5:**
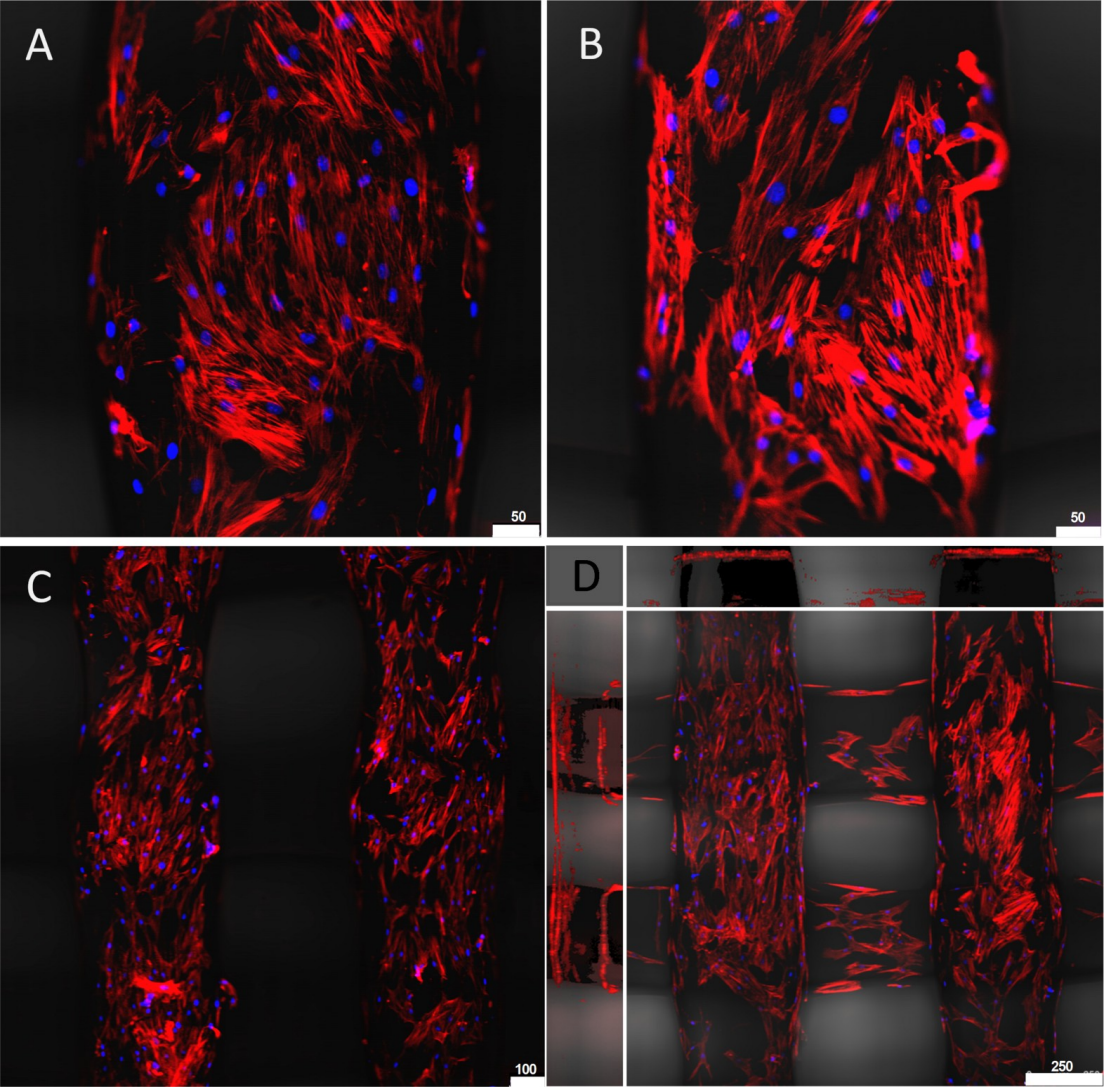
hMSCs imaged on PLA scaffold from the bioreactor. Cells underwent a three-day prime at 1.5% oxygen and were then cultured out for seven days. Cells were then fixed in place and stained with phalloidin (red) and DRAQ5 (blue). A) and B) show hMSC on a single fiber. C) Low magnification showing hMSC coverage among parallel fibers. D) Projected Z-stack of fibers showing cell coverage. The center image shows the top view (XY projection). The top and sidebars show sideways projection (ZX and ZY).

To visualize the cells cultured on the surface of the fibers we used fluorescence microscopy, as it was too difficult to resolve the cells on the PLA matrix using phase-contrast light microscopy. Phalloidin combined with DRAQ5 staining gave us good resolution of the cells and allowed us to visualize how the cells were interacting with the substrate at a superficial level. Cells formed confluent monolayers towards the top center of the lattice sampling shelf by day seven of culture (Fig 5), showing similar morphology to hMSCs cultured on control PS plates.

**Fig 6:**
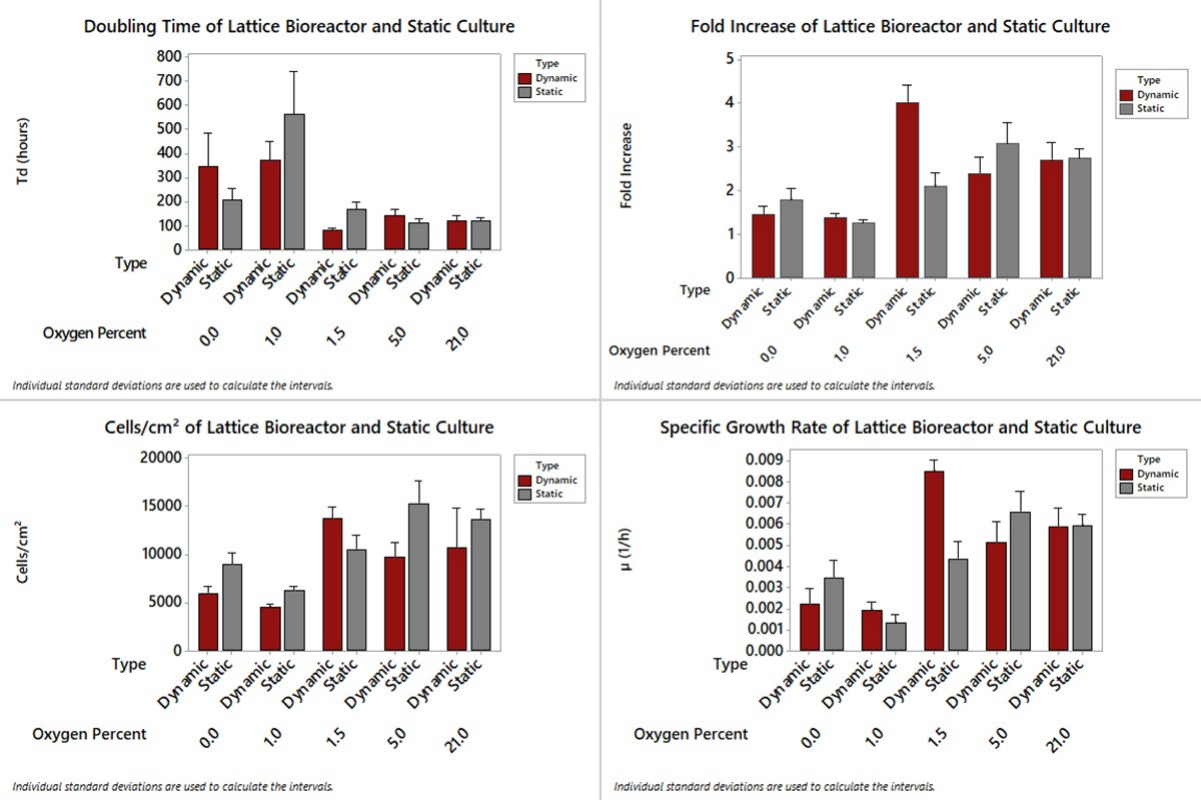
hMSC dynamic bioreactor culture vs static flask culture vs varying oxygen tension. Bioreactor and static cultures were harvested and compared on day seven. Static refers to T-75 tissue culture flasks and dynamic refers to bioreactor culture. A) Doubling time, B) Fold increase, C) Cell yield per cm^2^, and D) Specific growth rate of hMSC.

Normoxic reactor culture resulted in very similar doubling times as normoxic PS control cultures (Fig 6A). Because hMSCs are physiologically found in hypoxic conditions in vivo, oxygen tension was investigated as a means of increasing cell proliferation [41,42]. It was found that three-day priming in 1.5% O_2_ resulted in a four-fold increase in cell yield; double that of conventional flask culture methods tested (p<0.001) (Fig 6B). Normalized cell yield to surface area was 13,725 cells cm^-2^ at 1.5% O_2_ (Fig 6C). As mentioned, this in-situ conditioning resulted in a significant increase in the specific growth rate (0.0085 hr^-1^ ±0.0005) (Fig 6D).

**Fig 7:**
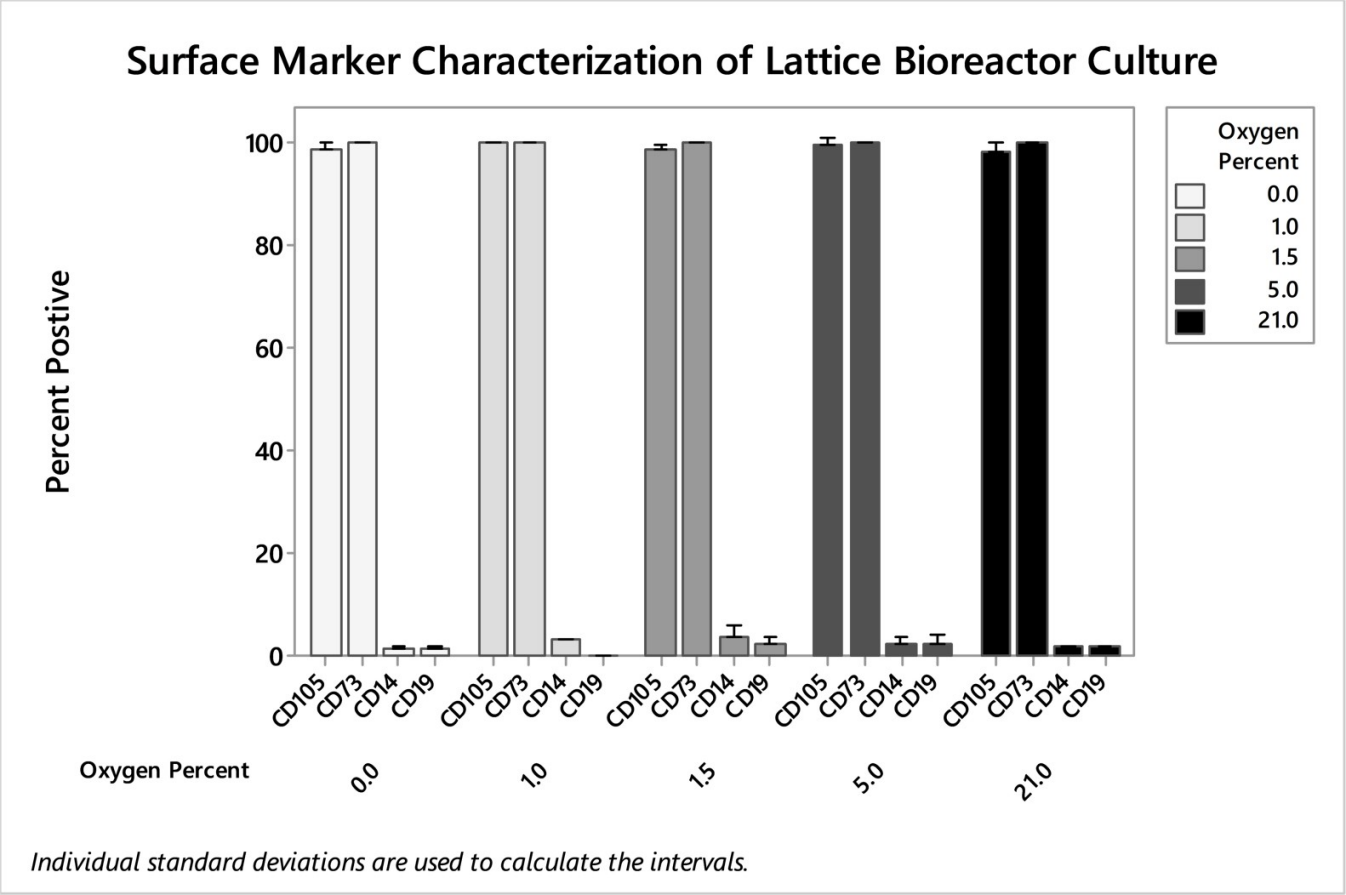
Flow cytometry of hMSC from bioreactors harvested day seven from varying oxygen tension. CD105, CD73, CD19, and CD14 stained cells were analyzed using flow cytometry. No significant differences were found in biomarker expression after preconditioning using 0.0%, 1.0%, 1.5%. 5.0%, and 21% gasses.

**Fig 8:**
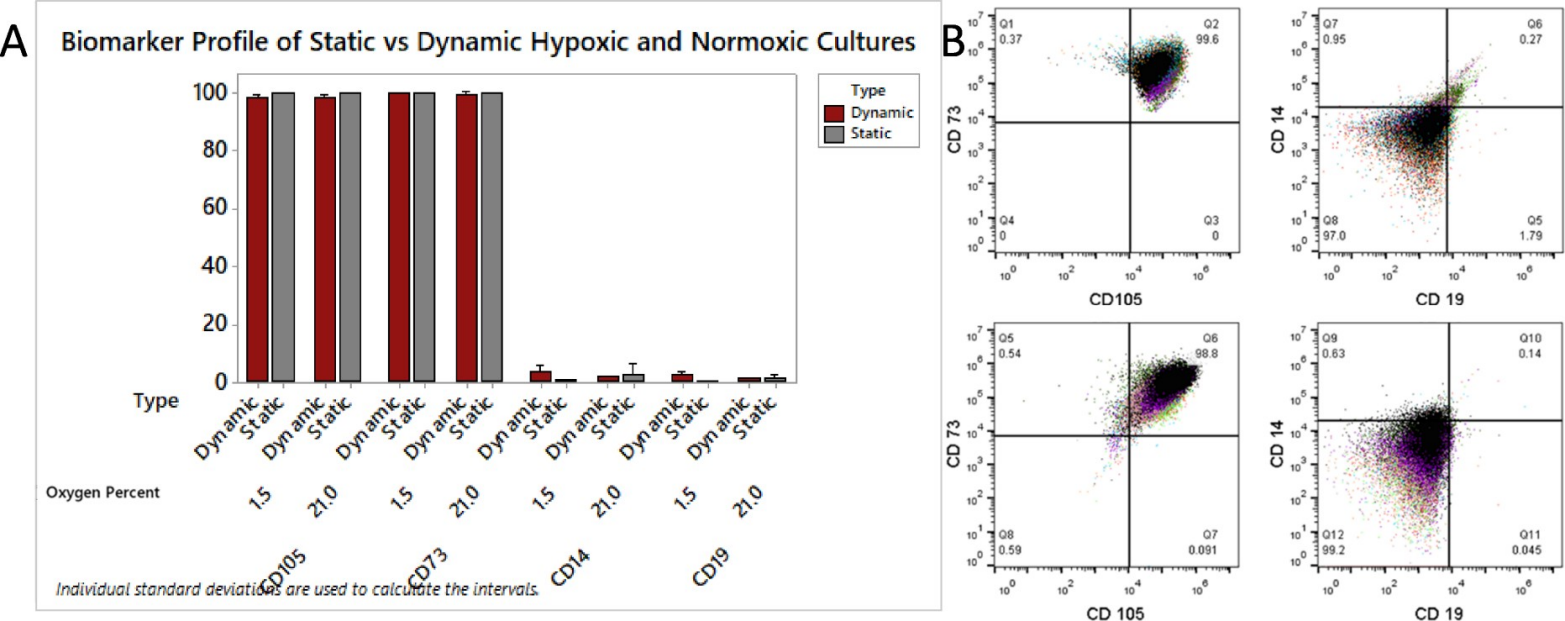
hMSC biomarker characterization using flow cytometry. Cells were cultured in both static and dynamic bioreactor conditions, and compared using CD105, C73, CD19, and CD14 staining. B) Overlaid flow cytometry image of 1.5% O_2_ primed hMSC cultures from day seven dynamic bioreactors showing positive markers and negative markers.

When lifted and analyzed via flow cytometry, it was found that cells cultured in the bioreactor retained their biomarker phenotype regardless of gas composition used for hypoxic treatment; ANOVA showed no significant difference in CD105 (p = 0.309), CD73 (p = 0.347), CD19 (p = 0.676), and CD14 (p = 0.523) biomarker expression (Fig 7). Thus, oxygen tension had a drastic effect on cell proliferation, but no effect on biomarker profile. Cultures primed at 0% and 1% (n = 3 for both conditions) produced statistically similar cell yields, and cultures primed at 5% and 21% oxygen produced statistically similar yields. Compared to control cultures on static tissue treated PS, the dynamic bioreactor culture on PLA produced higher purity MSCs according to ISCT standards. Harvested cells were over 98% dual CD105 and CD73 positive in reactor culture compared to 94% in static normoxic polystyrene culture (p = 0.005) (Fig 8A). There was no significant difference in the negative markers CD14 and CD19 under normoxic (n = 9) or 1.5% hypoxic conditioning (n = 6), and single populations of cells were harvested from bioreactors (Fig 8B).

### Differentiation potential

**Fig 9:**
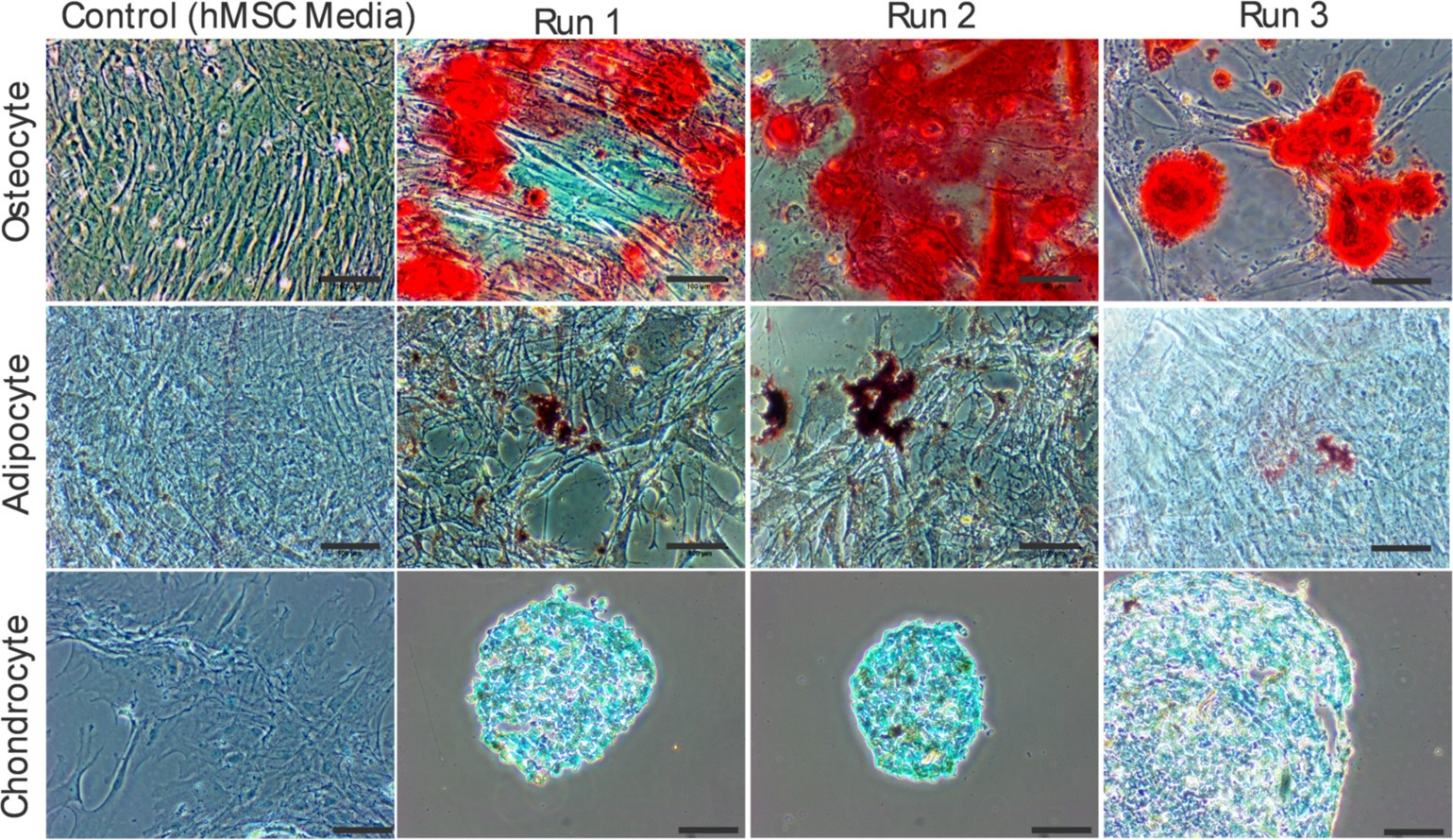
Stem cell induction. Cells harvested on day seven from the bioreactor and cultured out in respective differentiation media following specialized protocols. The top row shows osteocyte, the middle row shows adipocyte, and the bottom row shows chondrocyte inductions. After specified time points based on the individual ATCC protocols cells were fixed, stained, and imaged using light microscopy. Scale bars are 100μm. The first column shows stained negative controls of respective cultures.

To test differentiation potential per ISCT guidelines, osteocyte, adipocyte, and chondrocyte inductions were performed on stem cells harvested from the bioreactor after seven days of culture. After harvesting, cells were replated and cultured between 15 to 20 days in their respective defined ATCC differentiation media, after which cells were washed, fixed, and stained. After seven days in bioreactor culture and hypoxic conditioning, the cells retained their ability to differentiate into adipocytes, chondrocytes, and osteocytes (Fig 9). Flask-based negative control cultures cultured to hMSC ATCC guidelines were performed in parallel with the inductions. These control cultures showed no staining in uninduced hMSC controls cultured for 21 days in hMSC media.

## Discussion

By culturing hMSCs in this scaled-down bioreactor, we were able to double cell yield over conventional flask culture methods. It was found that cells cultured in this manner maintained high expression (>98% combined CD105+ and CD73+) of positive stem cell markers. PLA did not impact cell viability compared to cultures on polystyrene in either static or dynamic culture conditions. To compare, CFD of stirred tank reactors utilizing microcarriers report values of shear stress between approximately 1.0 to 5.0 dyne cm^-2^ (Table 1). Tubular and hollow fiber systems with similar laminar flow patterns report average values of 0.98 dyne cm^-2^ [43]. These values all fall within 0.02 to 9.0 dyne cm^-2^, a range shown to upregulate osteogenic genes and differentiation in hMSCs [15,44,45]. The system tested herein is an order of magnitude lower than this reference range, with a predicted average of 0.0029 dyne cm^-2^. The low calculated shear stress is reflected in the high percentage of pure hMSCs.

Surface biomarker profile of hMSCs did not change with oxygen percentage, as ranges tested were within physiological normoxia and treatment times were comparatively short to other hypoxic culturing [46]. These findings follow previous findings of oxygen tension promoting stem cell proliferation and stemness [47–50]. As mentioned previously, cells were most concentrated on the top of the fibers. Upon inspection, the cells seem to utilize only the top portion of the fibers, which would be a product of their static seeding. This would make the seeding density closer to 5,000 cells cm^-2^. This density matches that normal flask culture, but in an environment more conducive to hMSC culture.

The combination of hypoxic conditioning, gentle fluid movement, and substrate stiffness may better mimic their niche compared to static cultures. PLA printed by filament deposition has a Young’s modulus of 3.2 GPa, which falls in the range of elasticity of trabecular bone [37,51]. BM-hMSCs are normally harvested from the trabeculae of the iliac crest or the head of the femur. When these three factors are combined, it creates the normal niche for these stem cells, which could explain why the cells perform much better in the dynamic bioreactor culture condition compared to static PS flask culture.

hMSCs were also cultured on Cytodex-1 microcarriers as a benchmark. The doubling time of the tested spinner flasks was significantly longer compared to the lattice reactor. Spinner cultured cells were not characterized via flow cytometry, as even after an hour in TrypLE they did not lift from the Cytodex-1 beads. This problem of inadequate cell lifting has been noted before [52]. Conversely, cells cultured in the lattice system had no issue dissociating from the matrix using CDB and TrypLE. An added benefit of this system is that it requires less downstream purification compared to microcarriers. Per UPS <788>, removal of microcarriers as particulate matter is recommended for injected products [7]. To mitigate the added step of microcarrier removal, some microcarriers are themselves digested by enzymes. However, the byproducts of microcarrier digestion are still a concern for final formulation and patient administration [53]. Thus, systems using microcarriers for hMSC therapies would require additional inertial separation or straining and filtration steps to remove microcarriers from cells after dissociation, adding complication and potentially decreasing overall yield through [54,55]. Furthermore, filtration and straining have been shown to decrease the viability of harvested cells [55]. In our system, this purification step is more robust, as cells can be washed in place and lifted with reduced process-related impurities after dissociation. Another noted benefit of using this 3D printed structure is that unlike particulate from microcarriers, the degradation product of the PLA lattice is lactic acid [56]. This byproduct can easily be removed through buffer exchange, but can also be broken down naturally in the body and may not need removal from the cell product. Here we used TrypLE to dissociate the cells, but another potential dissociation technique for this reactor would be to use a thermoresponsive polymer. In future studies P(NIPAM) could be coated into the stationary PLA lattice, and cell lifting would be completed by simply dropping the temperature [57]. This can potentially eliminate the need for exogenous enzymes and washes, further decreasing downstream processes and subsequently increasing yield. Moreover, removal of the cells from the scaffold may not be necessary at all.

The scope of this study was to show that the cells harvested from a 3D PLA lattice were functional stem cells and could be lifted and further processed. However, cell detachment may not be necessary depending on the application. As PLA is biocompatible and similar in rigidity to cancellous bone, hMSCs can be expanded and differentiated in situ. This cell-laden 3D printed structure could then be implanted whole with the cultured cells attached as a therapy. Furthermore, most 3D printers can handle multiple types of polymers, and by choosing other materials or blends of materials, polymer rigidity can be tailored for the desired stem cell differentiation. Stiffer polymers like PLA, polystyrene, or polycarbonate (PC) can be printed using high temperature 3D printers and can be easily treated for cell adhesion for use in bone regeneration. Softer, more elastic materials like polyurethane have already been used for stem cell culture, and are also readily available materials for 3D printing [58]. Culture on elastic scaffolds, such as alginate encapsulation, can direct hMSCs to differentiate into chondrocytes and has already been used in established differentiation protocols [59,60]. This toolkit allows the user to customize the type of lattice to direct differentiation within this bioreactor if desired.

Another possible use of this system is to harvest secreted products. hMSCs are naturally adherent and will remain bound to the substrate, where they will release cytokines and exosomes of therapeutic interest into circulating media. Research into exosomes has already shown their usefulness in wound healing and inflammatory diseases [61–63]. The proposed lattice system can be run in perfusion, allowing simple harvest of the secretome while cells are held stationary in favorable conditions within the reactor.

## Conclusion

Here we show the successful use of a scale-down model of a novel suspended matrix bioreactor for the culture of hMSCs. Cells adhered well to PLA lattice and were able to form monolayers similar to conventional 2D culture techniques. A combinatory effect of low oxygen tension and slow recirculation rate of 0.25mL min^-1^ through the suspended lattice-based culture resulted in higher cell yields compared to conventional expansion systems, including static T-flasks and microcarrier-based spinner flasks. 1.5% O_2_ gas had the best overall fold expansion, resulting in a four-fold increase in overall cell yield. The cells were easily dissociated from the reactor and showed excellent stem cell biomarker expression. hMSCs also retained their ability to differentiate into bone, cartilage, and fat cells. While future studies must focus on scaling this process to larger bioreactors, with this work we have shown the validity of suspended matrix bioreactor systems for high purity hMSC production.

## Supporting information

S1 Fig Lattice dye testing. Figure showing modeled dye infiltration into the matrix at normal operating velocity (top row) to benchtop dye testing images at corresponding time points (bottom row).
(TIF)

S2 Fig Cells on cellulosic. Image of cells grown for seven days in bioreactor culture on cellulosic-based scaffolding. Scale bars are 100μm.
(TIF)

S3 Fig Comparison of culture methods. Culture method vs doubling time of hMSCs. Static cultures were grown in T-75 flasks according to ATCC guidelines. Spinner cultures used Cytodex-1 microcarriers in spinner flask. Dynamic culture used PLA lattice as per the described methods.
(TIF)

S1 File Novel low shear 3D bioreactor for stem cell culture. (XLSX)
