# Supplementary figures and images for: Novel low shear 3D bioreactor for high purity mesenchymal stem cell production
Source: PLoS One. 2021 Jun 16;16(6):e0252575. doi: 10.1371/journal.pone.0252575 (PMC8208585; doi:10.1371/journal.pone.0252575)

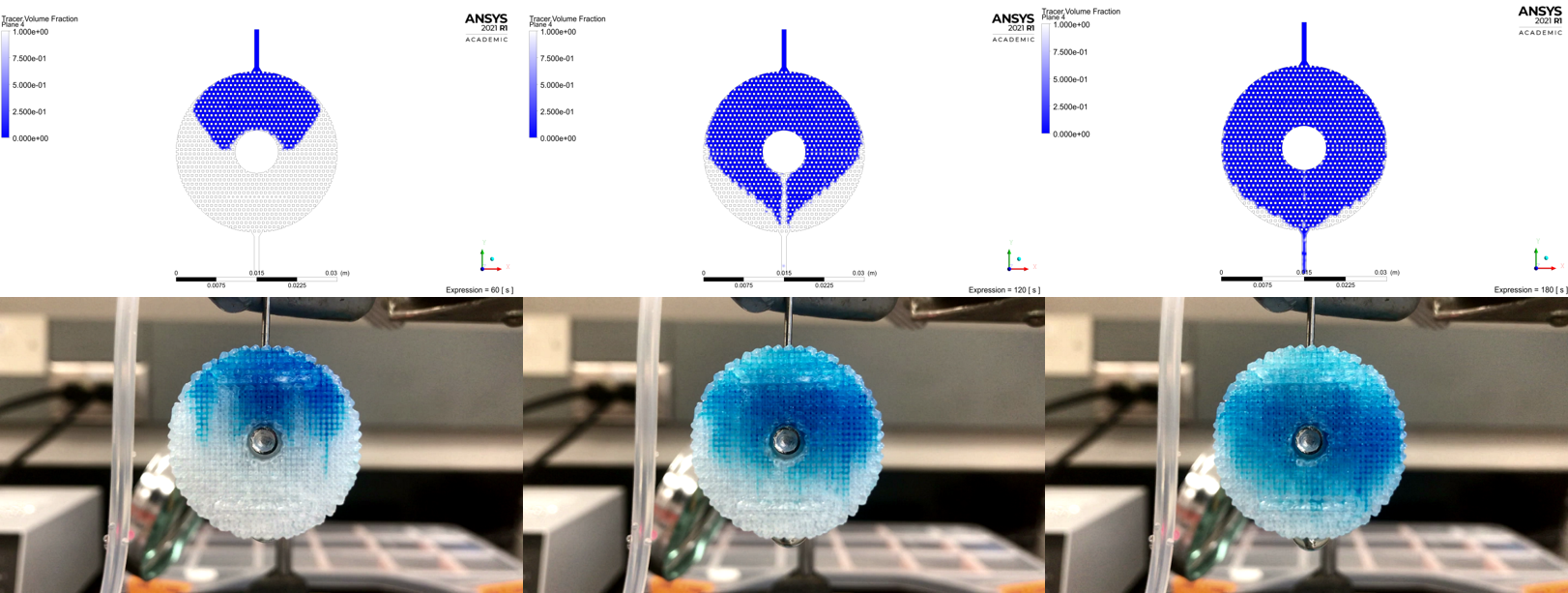

Supplement: S1 Fig — Figure showing modeled dye infiltration into the matrix at normal operating velocity (top row) to benchtop dye testing images at corresponding time points (bottom row). (TIF) [file pone.0252575.s001.tif]

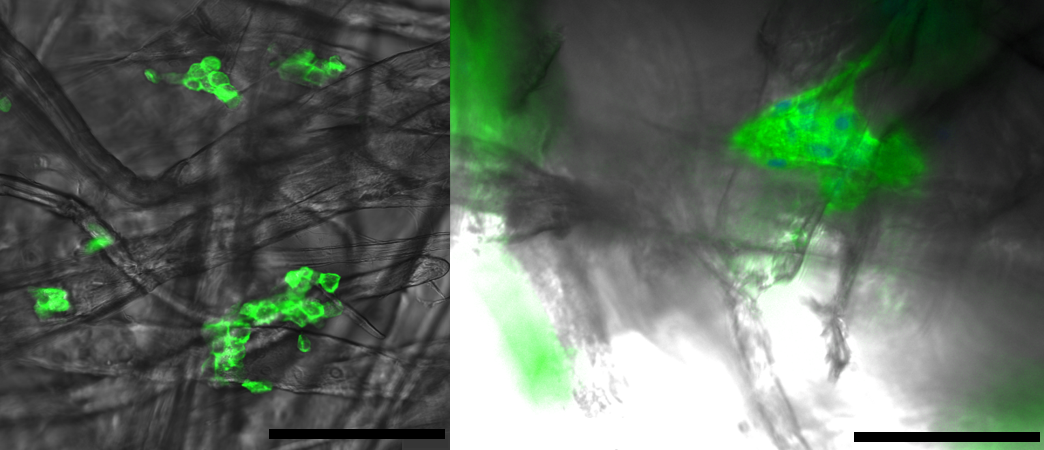

Supplement: S2 Fig — Image of cells grown for seven days in bioreactor culture on cellulosic-based scaffolding. Scale bars are 100μm. (TIF) [file pone.0252575.s002.tif]

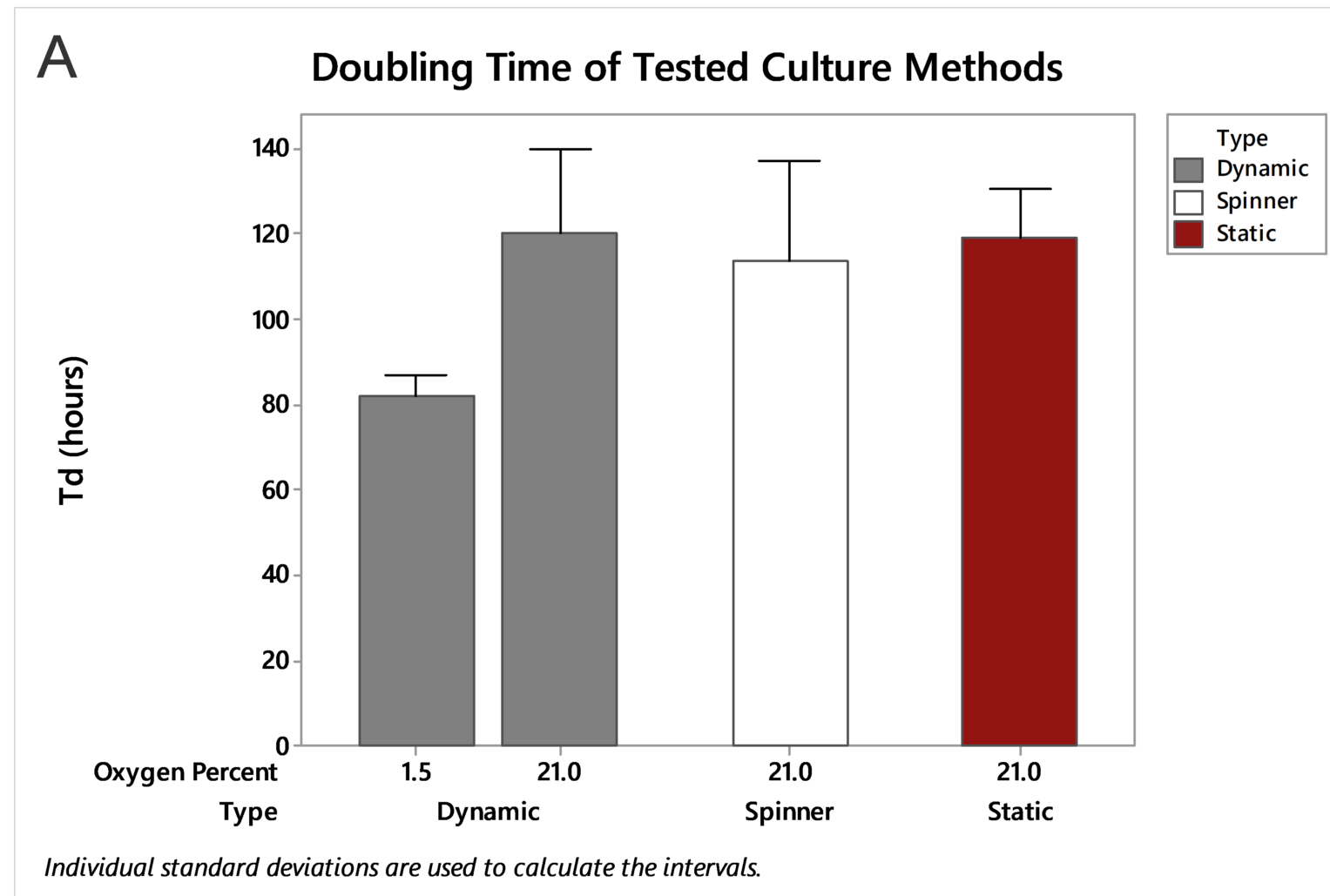

Supplement: S3 Fig — Culture method vs doubling time of hMSCs. Static cultures were grown in T-75 flasks according to ATCC guidelines. Spinner cultures used Cytodex-1 microcarriers in spinner flask. Dynamic culture used PLA lattice as per the described methods. (TIF) [file pone.0252575.s003.tif]
